# Phthisis with Perforation of the Bowel

**Published:** 1908-05-30

**Authors:** 


### Medicine.

### PHTHISIS WITH PERFORATION OF THE BOWEL.

The following case illustrates a number of important points in regard to phthisis, amongst others being the following:—

1. That it is extremely difficult to be sure that a person's lungs are healthy even when a very careful examination of the chest has been made by more than one observer.

This is of particular importance in connection with life insurance. The physical signs that one obtains on examining a chest depend almost entirely upon the physical condition of the superficial layer of lung tissue. If this superficial layer is healthy there may be quite extensive mischief beneath it, and yet neither percussion nor the use of the stethoscope will detect it. This has again and again been observed in cases of lobar pneumonia, for example; a patient may have every symptom of pneumonia, and the diagnosis may be perfectly obvious, even though there is not a single abnormal physical sign in either lung; the reason being that the pneumonic process in such a case has not reached the surface, and the physical signs obtained are those of the normal lung around the consolidated part. The same thing is illustrated in a different way in other cases; an examination made in the morning may reveal no abnormal physical signs at all, whereas when the examination is repeated an hour or two later there may be loud bronchial breathing and pectoriloquy in a part where in the morning there were normal voice sounds and vesicular murmur. This means that in the morning there was still a rim of normal lung between the stethoscope and the hepatised pulmonary tissue; by the afternoon the pneumonic process has extended into this rim of normal lung, rendering it pneumonic like the lung beneath it, and now that the consolidation has reached the surface the typical abnormal physical signs have suddenly appeared.

In precisely the same way, if there is a large phthisical cavity near the apex of the lung, with normal pulmonary tissue all round it, the physical signs obtained will be the normal ones from the normal lung tissue at the surface, and not the abnormal ones from the deep-seated cavity. This has been tersely expressed by the aphorism: "There are no such things as the physical signs of a cavity; there are only the physical signs of a cavity *with solid walls.*"

The post-mortem examination upon the case to be described showed that there were old cavities in the lungs which must have been present at the time when the patient's pulmonary condition was stated to be perfectly sound by two independent examiners.

2. That in the diagnosis of early phthisis symptoms are quite as important as physical signs.

The patient had not suffered from all the classical symptoms of phthisis, but a year previous to the time when the lungs were pronounced healthy he had had "pneumonia" with blood-stained sputum; and never since that time had he been free from cough, whilst at the same time, having lost weight during the "pneumonia," he had not regained it as he should have done. If night sweats had been added to his other symptoms the picture would have been complete.

There was no sputum that could be examined for tubercle bacilli, or otherwise the diagnosis would have been certain, even in the absence of abnormal lung signs.

3. That a sea life is no guarantee against phthisis, even when the conditions are as favourable as they are when the person is captain of the ship.

The patient had long been at sea; his symptoms first developed when he was at sea; and it was during his last cruise that the acute outburst of the tuberculous process that led to his death occurred.

This is a point of much interest in connection with the sea voyages that used to be advocated for phthisical patients. They are almost certain to do harm rather than good, unless the patient has practically thrown off his lung trouble, and only goes to sea when he is strong again for the sake of gaining additional strength.

4. That in cases of phthisis it is very uncommon to get general tuberculosis; the tubercles in consumption are confined almost always to the lungs, the larnyx, the intestines, and the kidneys. The larnyx and the intestines become infected from the sputum which has not been rapidly expectorated, but has first rested in the throat and then has been swallowed. The kidneys seldom become involved in these cases, but occasionally they do, probably during the process of elimination of some of the tubercle bacilli in the urine. From the kidney region it is not very rare for the vesiculæ seminales and the vasa deferentia to be affected too.

In this case the tuberculous process was extreme in the lungs, the larnyx, and the bowel, but it was limited to these, as is usually the case.

5. That when there is tuberculous laryngitis in a case of phthisis it is almost a certainty that there is tuberculous ulceration of the intestine also.

This is a fact which is very fully confirmed by post-mortem examinations. In the great majority of the cases there are no clinical symptoms pointing to the ulceration of the bowel during life, and yet when one makes a large series of autopsies in these cases one finds that there is tuberculous ulceration of the intestine in every single case in which there is any decided ulceration of a similar nature in the larnyx; and this no matter whether there have been bowel symptoms during life or not.

The case we give is rather exceptional in having exhibited so much diarrhœa. The latter should theoretically be present in every case of the kind, but in practice it is found that there is neither diarrhœa nor constipation to complain of, even when there may be quite extensive tuberculous ulceration of the intestine. Blood per rectum is a still rarer symptom of the trouble. The diagnosis has to be made without the help of symptoms, and if there is tuberculous ulceration of the larynx one may be perfectly certain that there is tuberculous ulceration of the bowel also.

6. That perforation of a tuberculous ulcer of the intestine, though not common, is a well recognised mode of termination in cases of advanced phthisis.

The reason why such perforation is not more common is that the process of tuberculous ulceration is a slow one; and hence there is nearly always time for inflammatory thickening to occur upon the serous surface of the bowel as fast as the tuberculous process burrows down from the submucous coat. This reason is precisely similar to that which prevents pneumo-thorax from being more common when there are large ulcerating vomicæ in the lung, and the pleura reacts and becomes thickened or adherent over the pulmonary lesion; otherwise pneumo-thorax would be an everyday occurrence. But, just as a sudden advance in the ulcerative process in a lung vomica may eat a hole through the thickened pleura and cause a pneumo-thorax, so may a sudden exacerbation of the intestinal ulceration eat a hole through the thickened peritoneum at the base of the ulcer. The result is either a local abscess, or else suppurative peritonitis as in this instance.

Some idea may be obtained of the frequency with which perforation of the bowel occurs as a terminal phenomenon in bad phthisis when one finds that at a large hospital where 650 post-mortem examinations are made annually, including deaths from all causes, from infantile marasmus to cut throat, there are from one to three cases of general peritonitis due to perforation of a tuberculous ulcer of the intestine every year. The condition is, as regards rarity or the reverse, about on a level with fatal pneumo-thorax, roughly speaking.

7. That when the patient is already very ill from phthisis, the occurrence of perforation of the bowel and general suppurative peritonitis may cause so few additional symptoms that they may not be noticed clinically at all.

In the case in question, had it not been for the post-mortem examination, it would not have been known that there was any general peritonitis.

This is precisely similar to what may be seen in typhoid fever, and in each case it is an example of the Weber-Fechner law. To produce a given increase in a sensation requires, not a fixed increase in the stimulus, but an increase in the latter amounting to a definite proportion of the already existing stimulus. For instance, if one listens to a watch ticking in the stillness of the night, it sounds very loud; but if one listens to the same watch when one is being rattled through a tunnel in a train, one can hardly hear it, if at all. Yet it is the same watch ticking in each case. Just in the same way, the sudden occurrence of general peritonitis in a perfectly healthy man gives rise to extreme and urgent symptoms; but if a similar general peritonitis occurs in a patient who is already as ill as he can be from something else, such as acute extensive phthisis, or typhoid fever, it is almost impossible for him to exhibit any further symptoms than he is already doing, and the occurrence of even so grave a complication as suppurative peritonitis may pass almost, or quite, unnoticed. This was the case here; and the same thing may be observed in connection with pneumo-thorax in other cases; if the patient is already very ill, pneumo-thorax may arise without causing any clinical symptoms to attract attention to it.

8. Lastly, that it is wonderful how little breathing lung may just suffice to carry on existence.

The patient had no useful lung anywhere in either upper lobe; practically none in the right lower lobe; a little at the anterior margin of the middle lobe; and about half of the left lower lobe. Altogether he had less than a single lobe left to breathe with out of both lungs. The importance of this in connection with lobar pneumonia is obvious. It is extraordinary to see how much lung may be consolidated on both sides of the chest in lobar pneumonia, in cases which —none the less—may recover if they are otherwise healthy and young.

The patient whose case serves to illustrate all the above points was a man aged 36, whose occupation had always been upon the sea, and he was now captain of his ship. He was married to a robust and healthy wife, and he had two children who were strong and well. There was no definite family history of tuberculosis.

The history was that he had for some years past been subject to attacks of hoarseness and "ulcerated throat" in the winter time; but he had regarded this as simply due to exposure to fogs and inclemencies of weather. During the summer, a year before he came under observation in hospital, he had an illness which he called "pneumonia;" he was abroad at the time, and the precise nature of the attack is obscure. He was laid up with this for a month, and in the earlier part of the attack his sputum was blood-stained. He got much better, but he did not regain his weight, and he had ever since been troubled with a cough without expectoration.

Being dissatisfied with his condition, he was examined by two medical men in consultation, with a view to deciding whether he should go the next cruise with his ship or follow some other course of life. He looked swarthy, and tanned, and well. His chest was big and broad, with a tendency to emphysema. No single abnormal physical sign could be detected in either lung; every system seemed normal. This was in May, and in November he was dead. He was advised to go with his ship, and he did so. On his return he was distinctly thinner, and it was now possible to hear crackling rales at both apices when he coughed. He got rapidly weaker, and took to his bed in August. He stayed there till the end, running the ordinary course of an acute phthisis. Throughout the last four months of his life there was very troublesome diarrhœa, and increasing huskiness of his voice. His spirits remained good, notwithstanding the downhill progress of his general condition. The abnormal physical signs in the lungs advanced until every lobe was known to be involved. The pulse rate was 120 for a long time before death, and there was considerable pyrexia every evening. There was no sudden ending to his life; the patient seemed to get just gradually weaker and weaker, and finally ceased to exist. At the post-mortem examination there was no lardaceous change in any organ. It will suffice to describe the lungs, larynx, bowel, and peritoneum, for the other parts were approximately normal.

The lungs had extensive adhesions over their pleural surfaces. The left upper lobe contained a very large old cavity the size of a golf ball, crossed by many bridles. The lung tissue left around the cavity was entirely fibro-caseous. The right upper lobe contained two old thick-walled cavities of smaller size than that in the left; and the rest of this lobe was crowded with crude tubercles and areas of caseous pneumonia. There was no cavitation in either of the other three lobes, but in each there was very extensive caseation, and innumerable grey tubercles, affecting nearly the whole of the middle lobe, the whole of the right lower lobe, and the upper half of the left lower lobe. The least affected portion of lung was the lower half of the left lower lobe, and even this was not free from scattered grey tubercles. The larynx exhibited extensive and typical tuberculous ulceration.

The peritoneal cavity was full of thick purulent fluid, which had clearly been present for a day or two at least. On examining the intestine, a large number of tuberculous ulcers were found in it. The first was 11 feet beyond the duodenum, and there were twenty-two large transverse and irregular ulcers between this and the ileo-cæccal valve. The large intestine was crowded with tuberculous ulcers; the mucosa of the cæcum was riddled with them, and thence they extended all the way to the sigmoid flexure, becoming less numerous as their distance from the cæcum increased. There were tuberculous ulcers in the vermiform appendix. Two of the ulcers had perforated; apparently the older hole was in the base of one of the ulcers just above the ileo-cæccal valve; the second was in the beginning of the sigmoid flexure.

It was impossible to say precisely when the perforation, and its consequent suppurative peritonitis, took place, but it was clear that it must have been at least a day or more before death. The peritonitis was, as has been stated, a surprise at the autopsy, for there had been no clinical indication of it at all.

